# Video-Assisted Thoracoscopy For Penetrating Cardiac Box Injury in Stable Patients

**DOI:** 10.21470/1678-9741-2020-0361

**Published:** 2021

**Authors:** Eric E. Vinck, Eduardo Posada Ángel, Rodolfo V. Barrios, Stella I. Martínez, Carlos A. Arias, Juan C. Garzón, Tjark Ebels, Sergio A. Alzate, Alexander Fernández

**Affiliations:** 1 Department of Cardiovascular Surgery, Cardio VID Clinic - Pontifical Bolivarian University, Medellín, Colombia.; 2 Department of Surgery, Pontificia Universidad Javeriana, Bogotá, Colombia.; 3 Department of Thoracic Surgery, El Bosque University, Bogotá, Colombia.; 4 Department of Cardiovascular Surgery, Central Military Hospital, Bogotá, Colombia.; 5 Department of Thoracic Surgery; Fundaciόn Cardioinfantil, Bogotá, Colombia.; 6 Department of Cardio-thoracic Surgery, University Medical Center Groningen, Groningen, Netherlands.; 7 Department of Cardiovascular Surgery, Fundación Clínica Shaio, Bogotá, Colombia.

**Keywords:** Wounds, Gunshot, Trauma Centers, Thoracic Injuries, Wounds, Penetraing, Pericardial Window Techniques, Data Management

## Abstract

**Introduction:**

In high-volume trauma centers, especially in developing countries, penetrating cardiac box injuries are frequent. Although many aspects of penetrating chest injuries have been well established, video-assisted thoracoscopy is still finding its place in cardiac box trauma and algorithmic approaches are still lacking. The purpose of this manuscript is to provide a streamlined recommendation for penetrating cardiac box injury in stable patients.

**Methods:**

Literature review was carried out using PubMed/MEDLINE and Google Scholar databases to identify articles describing the characteristics and concepts of penetrating cardiac box trauma, including the characteristics of tamponade, cardiac ultrasound, indications and techniques of pericardial windows and, especially, the role of video-assisted thoracoscopy in stable patients.

**Results:**

Penetrating cardiac box injuries, whether by stab or gunshot wounds, require rapid surgical consultation. Unstable patients require immediate open surgery, however, determining which stable patients should be taken to thoracoscopic surgery is still controversial. Here, the classification of penetrating cardiac box injury used in Colombia is detailed, as well as the algorithmic approach to these types of trauma.

**Conclusion:**

Although open surgery is mandatory in unstable patients with penetrating cardiac box injuries, a more conservative and minimally invasive approach may be undertaken in stable patients. As rapid decision-making is critical in the trauma bay, surgeons working in high-volume trauma centers should expose themselves to thoracoscopy and always consider this possibility in the setting of penetrating cardiac box injuries in stable patients, always in the context of an experienced trauma team.

**Table:** 

Abbreviations, acronyms & symbols	
**CXR** **IVC** **OR** **VATS** **US**	**= Chest X-ray** **= Inferior vena cava** **= Operating room** **= Video-assisted thoracoscopic surgery** **= Ultrasound**

## INTRODUCTION

The first suture in a wounded heart in Colombia was performed in Bogotá in 1914 ^[^^1^^]^. Due to its internal conflicts, Colombia has always been known for its battlefield-like medicine and trauma resulting in an important socioeconomic burden. However, surgeons have adopted minimally invasive approaches to trauma in an evolving fashion ^[^^2^^]^. The aggressive approach to chest trauma in Colombia has a lot to do with the violent nature of these events and weapon dimensions. In Colombia, many knife wounds result from large blades, causing penetrating wounds deeper than the superficial entry wound may lead to believe. In developed countries, the number of penetrating cardiac injuries by gunshots outnumber stab wounds by a ratio of 2:1 ^[^^3^^]^. In developing countries such as Colombia, however, stab wounds are more frequent.

Case series in the acute scenario of trauma have reported promising results with video-assisted thoracoscopic surgery (VATS) for hemothorax evacuation, pulmonary wedge resections due to lung laceration, evaluation and repair of chest wall injuries, and repair of diaphragm injuries without the need for conversion to open techniques or reoperation ^[^^4^^-^^6^^]^. In the trauma scenario, many factors must be considered since surgeon skill and patient condition define the surgical approach. In high-volume trauma centers, especially in developing countries, penetrating cardiac box injuries are frequent. Although many aspects of penetrating chest injuries have been well established, video-assisted thoracoscopy is still finding its place in cardiac box trauma and algorithmic approaches are still lacking. The purpose of this manuscript is to provide a review establishing a streamlined approach to penetrating cardiac box injuries in stable patients using VATS. This algorithmic approach was driven by the need to standardize the minimally invasive thoracoscopic treatment for these life-threatening cases due to the high volume of trauma patients seen in Colombia.

## METHODS

Literature review was carried out using PubMed/MEDLINE and Google Scholar databases to identify articles describing the characteristics and concepts of penetrating cardiac box trauma treated with video-assisted thoracoscopy. Articles included were those that show characteristics of penetrating cardiac box injuries, cardiac tamponade, cardiac ultrasound, and indications and techniques of pericardial windows. Papers on video-assisted thoracoscopic approaches to cardiac box injury in stable patients were reviewed with an emphasis on Colombian approaches.

## RESULTS

### The Cardiac Box

Variations in the anatomical location of what is known as the cardiac box or “precordial” area are found in the literature. In Colombia, we use the area defined by Sauer and Murdock, limited superiorly by clavicles and jugular notch in the sternum, laterally between the left anterior axillary line and the right midclavicular line, and inferiorly by the immediate upper epigastrium ^[^^7^^]^. Many regions of the world also slightly expand the limits of this zone according to their own statistics and trauma characteristics. For example, some consider the epigastric area as precordial and others the supraclavicular area depending on the trajectory and angle of the injury.

### Stable, Unstable & Signs of Tamponade

For trauma patients, the classic cut-off values for classifying a patient as hemodynamically unstable are systolic blood pressure <90 mmHg; in many cases, it is also accompanied by a mean arterial pressure <60 mmHg, along with a heart frequency of >100 beats per minute, capillary refill time >2 seconds and, in some cases, altered level of consciousness ^[^^8^^]^. In general, trauma patients are considered unstable if there are clinical signs of cardiac tamponade or hypotension, regardless of heart rate and consciousness. In 1935, Dr. Claude Beck described the classic triad of cardiac tamponade: hypotension, muffled heart sounds, and jugular venous distention ^[^^9^^]^. Other signs of cardiac tamponade include restlessness, dyspnea, tachypnea, chest pain, sinus tachycardia, Kussmaul’s sign and *pulsus paradoxus* ^[^^10^^]^. Cardiac tamponade can also produce specific changes in the electrocardiogram, such as low QRS amplitude and electrical alternans ^[^^11^^-^^13^^]^. Although the Beck's triad is classically an academic tool, it is seen in only 10-30% of tamponade cases ^[^^14^^]^. Therefore, although these signs are not always constant, they provide a reliable representation of cardiac tamponade and, when present, have a sensitivity of 94% and specificity of 100%. ^[^^15^^,^^16^^]^.

### Pericardial Ultrasonography in Penetrating Cardiac Box Injury

Whether a cardiac ultrasound (US) is positive or negative in trauma depends on certain imaging characteristics. These include: amount of intrapericardial effusion (cm), wall/septal deviation, inferior vena cava (IVC) collapsibility, right chamber diastolic movements, and visceral to parietal pericardial separation ^[^^17^^]^. Since the normal pericardial fluid physiology allows for up to 50 cc of pericardial fluid, 10 mm of pericardial separation in the US may be considered normal. The European Society of Cardiology’s guidelines classify the pericardial fluid accumulation as mild (<10 mm), moderate (10-20 mm), and severe (>20 mm) ^[^^18^^,^^19^^]^. As a result, the patient’s height should be taken into consideration. Colombians are generally shorter than Europeans, therefore 15 mm is more significant in Latin Americans than in North Americans, for example ^[^^20^^]^ (Table 1). In the context of trauma, if the pericardial US exhibits the presence of significant pericardial fluid, it should be considered positive and be managed with surgical exploration.

**Table 1:** Ultrasonographic signs of cardiac tamponade.

Anatomy	Characteristics
Pericardial separation	>1.0 cm (relative to the patient's height)
IVC	Dilated
Wall/septal deviation	Deviated to the right
Right chamber behavior	Paradoxical movements

IVC=inferior vena cava

Initial Approach Using Chest X-Ray and Ultrasound

Since chest X-ray (CXR) and ultrasound (US) are widely available, non-invasive, cost-effective and can be evaluated in an extremely rapid manner, they are the initial exams to be performed in a hemodynamically stable chest trauma patient. According to the ultrasonographic and radiographic findings, the patient may be placed into one of four categories (Table 2).

**Table 2:** Initial approach using CXR and US in hemodynamically stable patients.

CXR	US	Approach
Negative	Negative	Continuous monitoring, observation and reevaluation with repeated imaging at 3-6 hours
Positive	Negative	Chest tube
Negative	Positive	Pericardial window
Positive	Positive	VATS pericardial window, open surgery according to clinical judgment and pericardial window findings

CXR=chest X-ray; US=ultrasound; VATS=video-assisted thoracoscopic surgery

Classification of Penetrating Cardiac Box Injury

Multiple classifications of precordial trauma can be found in the literature, including, but not limited to, Ivatury in 1987 ^[^^21^^]^, Saadia in 1994 ^[^^22^^]^, and the scaling system for organ specific injury of the American Association for the Surgery of Trauma in which cardiac injury is the section III. Traditionally, and for academic purposes in Colombia, penetrating cardiac box “precordial” trauma has been classified into three types. Table 3 summarizes and describes the treatment according to the classification from Bogota which was adapted and modified from Ivatury ^[^^21^^]^.

**Table 3:** Penetrating Cardiac Box Injury classification used in Bogota.

Classification	Characteristics	Traditional intervention	Treat as
Type 1	Stable/no signs of tamponade	CXR/pericardial US	Pericardial window according to US findings
Type 2	Unstable/signs of tamponade	Thoracotomy/sternotomy	Unstable
Type 3a	Unstable/patient goes into witnessed cardiac arrest	Thoracotomy/sternotomy	Unstable
Type 3b	Patient arrives in witnessed cardiac arrest	Thoracotomy/sternotomy	Surgeon's choice
Type 3c	Patient arrives in non-witnessed cardiac arrest	No intervention	No intervention

CXR=chest X-ray; US=ultrasound

### Positive Thoracoscopic Pericardial Window (Hemopericardium)

**Fig. 1:**
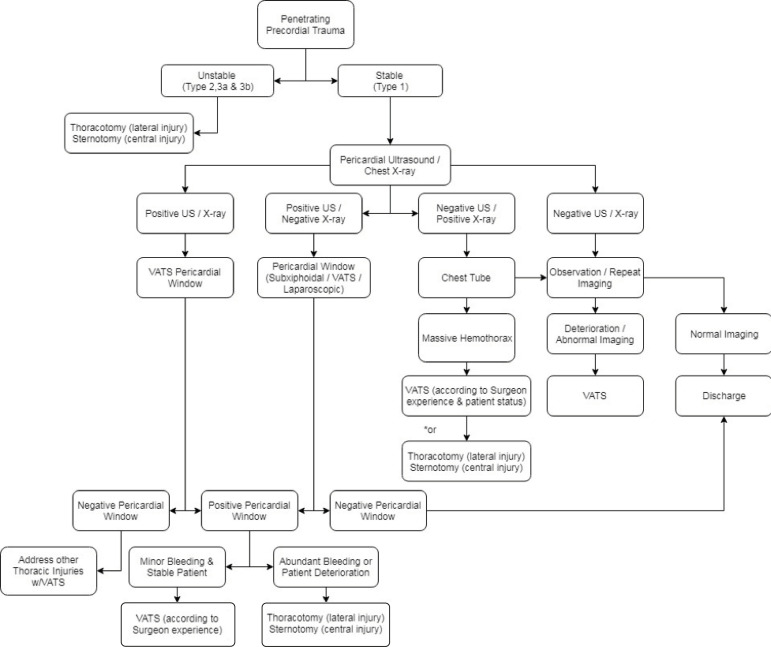
Algorithmic VATS approach to penetrating cardiac box injury for stable patients.

When a pericardial window is positive (hemopericardium), open surgery is mandatory. The current standard of practice when a “thoracoscopic” pericardial window is positive with hemopericardium is the conversion either to thoracotomy or sternotomy. In certain cases, pericardial bleeding or superficial cardiac bleeding may be minor and continuing with VATS may be appropriate if the patient remains stable. However, there is currently no recommendation in the trauma scenario about carrying on with VATS aside from a few case reports and small series ^[^^23^^-^^26^^]^. Although these reports show successful thoracoscopic treatment after positive pericardial windows, at this time we still cannot offer a universal recommendation for a thoracoscopic approach to treat positive pericardial windows, unless the surgeon has valuable experience with VATS, the patient is hemodynamically stable, and active bleeding is minor ^[^^24^^,^^26^^]^. Table 4 lists reports of VATS with positive pericardial windows ^[^^24^^,^^26^^]^. Figure 1 displays our recommended algorithmic VATS approach to penetrating cardiac box injury in stable patients used in Colombia.

**Table 4:** Reports of stable patients with hemopericardium managed with video-assisted thoracoscopy following penetrating cardiac box injury.

Author	Country	Year	Number of patients	Age	Hemodynamic stability	Wound/type of trauma	Surgical technique	Operative findings	Conversion to open surgery	Mortality
Andrade-Alegre^[^^25^^]^	Panama	2015	1	29	Yes	Stab wound in the right parasternal line	VATS + pericardial window	Hemopericardium	No	None
Correa^[^^24^^]^	Colombia	2016	1	22	Yes	Stab wound in the left precordial region	VATS + pericardial window + ventricle stitching	Hemopericardium, 1 cm wound in the right ventricle	No	None
Navsaria^[^^26^^]^	South Africa	2006	1	29	Yes	Anterior left-sided precordial wound	VATS + pericardial window	Hemopericardium, left ventricular contusion, pericardial bruising	No	None
Bucheli ^(not yet published)^	Cali, Colombia	2018	1	18	Yes	Transthoracic - trans-mediastinal GSW	VATS Pericardial window - bullet retrieval - Local Compression - pericardial lavage	400cc Hemopericardium, bullet lodged in the left atrium adjacent to pulmonary veins	No	None
		2020	1	60	Yes	Multiple Parasternal/precordial stab wounds	VATS Pericardial window - Local Compression- Hemostatic fibrin sealant	400cc Hemopericardium, Superficial infundibulum wound with minor bleeding	No	None

VATS=video-assisted thoracoscopic surgery

### Types of Pericardial Windows

#### VATS Pericardial Window

VATS pericardial window is the ideal approach when a patient has a positive CXR and/or a thoracoabdominal injury ^[^^27^^]^. The main advantage of VATS is the treatment of both thoracic and diaphragmatic injuries, especially hemothorax, if the pericardial window is negative. VATS pericardial window has been shown to be safe even under local anesthesia and sedation ^[^^28^^]^. However, this approach is easier if the patient has selective lung ventilation, anatomical free pleural space and availability of special video equipment ^[^^27^^-^^30^^]^.

#### Parasternal-Trans-mediastinal Pericardial Window

The anterior parasternal pericardial window is a relatively new approach to pericardial windows, described in Colombia in 2005, it shows great advantages in obese patients and women with large breasts ^[^^30^^-^^32^^]^. This approach offers the ability to easily convert the same incision into a thoracotomy and is usually advised when the chest injury is lateral.

#### Subxiphoid or Retrosternal Pericardial Window

The subxiphoid approach has been one of the most used techniques, first described in the early 1800s during the Napoleonic wars ^[^^33^^]^. This approach is suitable for central thoracic injuries that may end up in conversion to sternotomy through the same incision. Nonetheless, the subxiphoid approach presents technical difficulties in obese patients, patients with narrow subxiphoid angles, and in patients with previous subxiphoid pericardial windows ^[^^30^^]^.

#### Laparoscopic-Transdiaphragmatic Pericardial Window

In the scenario of a patient with a thoracoabdominal trauma in which abdominal organ damage or active bleeding, as well as pericardial or cardiac injury, need to be ruled out simultaneously, the transdiaphragmatic laparoscopic pericardial window is a suitable option in which both cavities can be evaluated through the same portal access ^[^^27^^,^^34^^-^^37^^]^. In patients with no suspicion of associated abdominal injury, a subxiphoid or thoracic (VATS/parasternal) approach is recommended.

## DISCUSSION

As surgical technology and innovations impulse the expansion of minimally invasive horizons, we should keep in mind our limits to avoid causing harm to patients who require a more aggressive care, especially in trauma. The newer generation of surgeons is exposed to endoscopic approaches much earlier; as a result, some younger surgeons sometimes feel more willing to use a VATS approach than open surgery. In the scenario of penetrating cardiac box trauma, the hemodynamic stability of the patient is the main decision-maker when it comes to choosing the type of procedure; in unstable patients with classifications 2, 3a and 3b, an open approach is mandatory; however, if the patient is stable (classification 1), a minimally invasive approach is an option. The attending surgeon should, therefore, be familiar with the specific institutional protocols to provide early care and to suspect cardiac injuries, and if he/she has experience with VATS, it can be performed in hemodynamically stable patients ^[^^34^^-^^37^^]^.

Finding hemopericardium during VATS pericardial window is life-threatening in the setting of penetrating injury. The five reports presented in Table 4 provide preliminary data that future research is merited for this specific group of patients. Many penetrating cardiac injuries do not require open surgery, and an initial diagnostic (thoracoscopic) approach may spare patients unnecessary interventions. In addition, in the hands of an experienced surgeon, even hemopericardium can be treated thoracoscopically, as long as the patient remains stable and the bleeding is minor. As a result, rapid identification and intraoperative decision-making are critical to separate those patients with true penetrating cardiac injuries and those with pericardial or superficial cardiac injuries that may be treated thoracoscopically.

Trauma centers in developing countries aspiring to develop minimally invasive trauma protocols should take many variables into consideration. A trauma team should consist of a multidisciplinary group capable of managing video-assisted thoracoscopy, OR personnel with experience in endoscopic approaches, well trained scrub nurses with knowledge of video equipment as well as open trauma surgery, and anesthesiologists with experience in managing one-lung ventilation and trauma. In 2017, Isaza-Restrepo et al. reported a series of 240 patients in Bogota with penetrating cardiac injuries and described the results of surgical treatment and mortality. Although they do not clearly describe an algorithmic approach to cardiac box injury, they provide a valuable analysis of the demographics of cardiac trauma in Colombia ^[^^38^^]^.

As in many regions of the world general surgeons are the first to face penetrating cardiac box injuries, we recommend that surgeons working in high-volume trauma centers train in video-assisted thoracoscopic approaches to offer a minimally invasive option to trauma patients. In addition, we recommend that cardiac and thoracic surgeons provide constant support and guidance to general surgeons in the treatment of chest trauma patients, since many may require corrective cardiac and pulmonary surgeries later. Penetrating cardiac box injuries continue to be a public health burden and a technically challenging surgical scenario due to the nature of these events, in which time is a critical issue and the surgeon’s skill and experience are key variables.

## CONCLUSION

During a positive VATS pericardial window, the surgeon should quickly determine whether the bleeding is pericardial or cardiac and, if so, whether the bleeding is minor. If the patient remains stable and the surgeon has experience in VATS, a thoracoscopic approach may be continued; otherwise, quick conversion to open surgery is mandatory. We recommend that surgeons working in the trauma bay expose themselves to VATS and always consider the possibility of performing minimally invasive procedures in the setting of penetrating cardiac box injury in stable patients, always in the context of an experienced trauma team.

**Table:** 

Authors' roles & responsibilities	
EEV	Substantial contributions to the conception or design of the work; or the acquisition, analysis, or interpretation of data for the work; final approval of the version to be published
EPA	Substantial contributions to the conception or design of the work; or the acquisition, analysis, or interpretation of data for the work; final approval of the version to be published
RVB	Substantial contributions to the conception or design of the work; or the acquisition, analysis, or interpretation of data for the work; final approval of the version to be published
SIM	Drafting the work or revising it critically for important intellectual content; final approval of the version to be published
CAA	Drafting the work or revising it critically for important intellectual content; final approval of the version to be published
JCG	Drafting the work or revising it critically for important intellectual content; final approval of the version to be published
TE	Drafting the work or revising it critically for important intellectual content; final approval of the version to be published
SAA	Drafting the work or revising it critically for important intellectual content; final approval of the version to be published
AF	Drafting the work or revising it critically for important intellectual content; final approval of the version to be published
